# Source files of the Carbohydrate Structure Database: the way to sophisticated analysis of natural glycans

**DOI:** 10.1038/s41597-022-01186-9

**Published:** 2022-03-30

**Authors:** Philip V. Toukach, Ksenia S. Egorova

**Affiliations:** grid.4886.2 0000 0001 2192 9124 N.D. Zelinsky Institute of Organic Chemistry, Russian Academy of Sciences, Leninsky prospect 47, Moscow, 119991 Russia

**Keywords:** Databases, Carbohydrates

## Abstract

The Carbohydrate Structure Database (CSDB, http://csdb.glycoscience.ru/) is a free curated repository storing various data on glycans of bacterial, fungal and plant origins. Currently, it maintains a close-to-full coverage on bacterial and fungal carbohydrates up to the year 2020. The CSDB web-interface provides free access to the database content and dedicated tools. Still, the number of these tools and the types of the corresponding analyses is limited, whereas the database itself contains data that can be used in a broader scope of analytical studies. In this paper, we present CSDB source data files and a self-contained SQL dump, and exemplify their possible application in glycan-related studies. By using CSDB in an SQL format, the user can gain access to the chain length distribution or charge distribution (as an example) in a given set of glycans defined according to specific structural, taxonomic, or other parameters, whereas the source text dump files can be imported to any dedicated database with a specific internal architecture differing from that of CSDB.

## Abstract (summary)

**Table:** 

Measurement(s)	scientific literature on natural glycans
Technology Type(s)	annotation of publications
Sample Characteristic - Organism	Bacteria • Archaea • Viridiplantae • Fungi

## Background & Summary

Glycoinformatics is a relatively new research branch, which provides the scientists with various means of accessing, processing and handling all sorts of carbohydrate-related data^1^. The broad usage of glycomic databases and associated software tools has been recently reported^2–8^. Similar to other data-related scientific branches, glycoinformatics heavily depends on high-quality data repositories. In the last decades, several such repositories have been developed. They include a historical CCSD project (CarbBank; contained more than 15,000 natural glycans before it was discontinued in 1996; the source of older data for most of the existing carbohydrate databases)^9^; Glycosciences.DB (contains the CCSD data supplemented with NMR spectra, 3D structures and analytical tools)^10,11^; UniCarbKB (contains eukaryotic glycans supplemented with NMR, MS and HPLC data)^12^; KEGG Glycan (glycan-related data from the Kyoto Encyclopedia of Genes and Genomes)^13^; Japan Consortium for Glycobiology and Glycotechnology (JCGG/ACGG collection of databases on glycoproteins and glycome-associated diseases supplemented with analytical data)^14^; and CSDB (the Carbohydrate Structure Database, see below)^15^, to name a few.

Successful application of any database depends not only on the quality and completeness of its data, but also on the capabilities and user friendliness of its interface. Thus, most of the chemical and biological databases, including carbohydrate ones, are equipped with a web interface. However, in many cases the source database files contain much more data than those accessible via the Internet, because the frontend interfaces and the backend tools behind them are usually designed to serve only the most popular and demanded user queries.

The Carbohydrate Structure Database (CSDB, http://csdb.glycoscience.ru/) is a free curated repository, which stores various types of data (structural, taxonomical, bibliographical, NMR spectroscopic, etc.) on glycans of bacterial, fungal and plant origins^15^. One of the most significant characteristics of CSDB is its completeness^15^. Currently, it provides a close-to-full coverage on bacterial and fungal carbohydrates up to the year 2020. The fungal coverage has been achieved in 2021 and has not been reported elsewhere.

CSDB is supplied with a web interface, which provides free access to the CSDB content and dedicated data analysis and simulation tools. These tools include coverage statistics, monomeric residue properties, multiparametric analysis of distribution of carbohydrate structural elements among taxa^16^, simulation of 1D and 2D NMR spectra^17–19^, NMR-based structure elucidation^20^, and structure translators to various carbohydrate and chemical notations^21^ and optimized atomic coordinates^22^. CSDB is integrated with a glycosyltransferase database (CSDB_GT), which currently covers GTs from the three most studied non-animal model species (*Escherichia coli*, *Saccharomyces cerevisiae*, *Arabidopsis thaliana*)^23–25^.

**Fig. 1:**
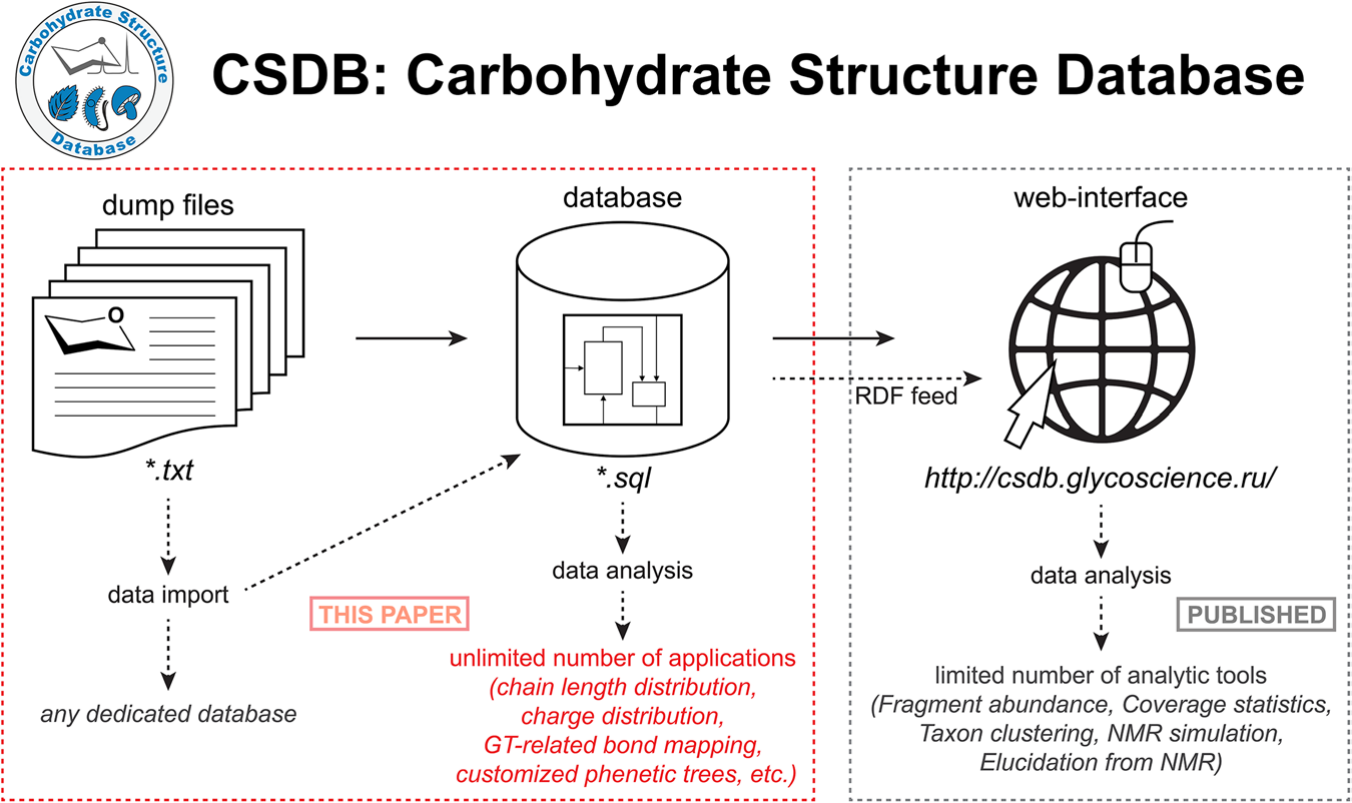
Source text dump files and SQL files for CSDB are reported in this paper. The CSDB web-interface, associated web tools, and RDF-ized data have been reported elsewhere^15,16,26,37^. Solid arrows represent immanent logic of the database; dashed arrows show inferred data flows upon usage.

According to user feedback, citing and access log analysis, the above-listed CSDB instruments are most demanded in routine research on natural carbohydrates. However, the number of these tools and the types of the corresponding analyses is limited, whereas the database itself contains data that can be used in a broader scope of analytical studies. For example, by using the existing database in an SQL format, the user can gain access to the chain length distribution or charge distribution in a given set of glycans, which can be defined according to specific structural, taxonomic, or other parameters. In their turn, the source text dump files can be potentially imported to any dedicated database with a desired internal architecture differing from that of CSDB (Fig. 1). In this paper, we present CSDB source text files (called dump files) and a self-contained SQL backup, and exemplify their possible application in glycan-related studies. By using these dump files, scientists can build dedicated databases suited for their particular scientific needs. The CSDB data can also be downloaded as an RDF feed generated within the GlycoRDF ontology^26^ for further import to an external triplestore.

## Methods

### Database architecture

**Fig. 2:**
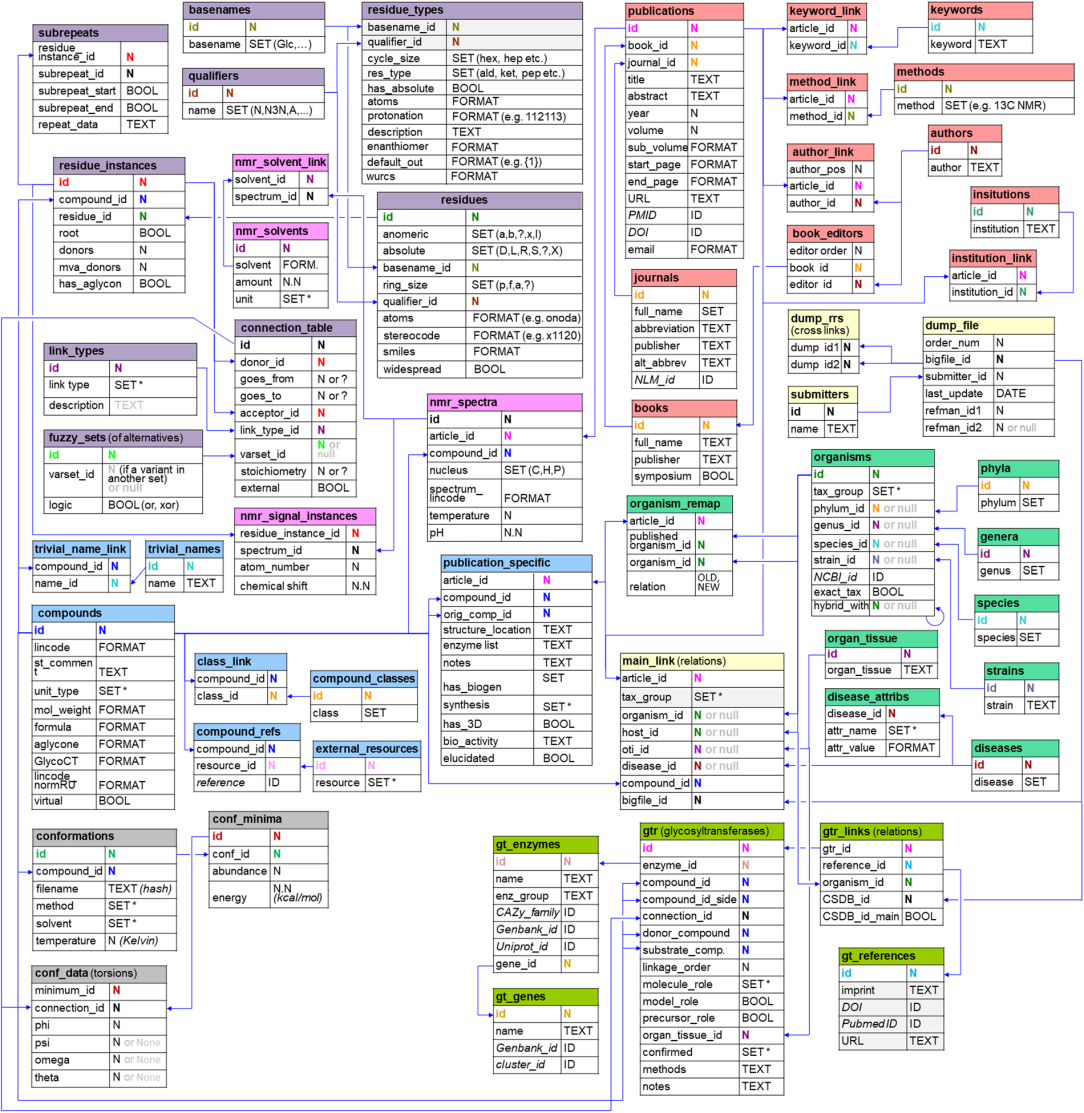
CSDB entity relationship scheme. In each table, the first column corresponds to the field, and the second column – to the data type: N, integer (the symbols of the same color correspond to the same indices connected by arrows in different tables); N.N, float; TEXT, text; FORMAT, formatted text, SET, controlled vocabulary term (see the main text); BOOL, boolean switch; ID, identifier in the external database. The color of the table headers reflects the data category: violet = molecular structure; cyan = compound as a whole; red = bibliography; pink = NMR data; green = taxonomy; olive = glycosyltransferases; grey = simulated conformations; yellow = main relations. The table meaning is explained in parentheses, where unclear from the table name (shown in bold). Blue arrows show one-to-many relations between the fields. Links to external resources are shown in italic; denormalized data are greyed.

CSDB stores data in a MySQL relational database. For structures, the connection table approach is used, where nodes are monosaccharide and other residues, and vertices are bonds with elimination of water. Relationships between the data from scientific publications and their indices are visualized in Fig. 2. The data are stored in database tables (see caption to Fig. 2). The following data categories are used (as reflected by the color of the table headers): molecular structure (violet); compound as a whole (cyan); bibliography (red); NMR spectra (pink); taxonomy (green); glycosyltransferases (olive); simulated conformations (grey); and main relations (yellow). The SET data type means a term from a controlled vocabulary, including large lists, such as monomer names, species, journal names, etc. Where not explained in the figure (marked with *), the following controlled vocabularies are implied:

- organisms.tax_group - bacteria, archaea, fungi, plant, protista, animal, mammal, human, etc.
- main_link.tax_group - the same as above (denormalized).
- conformations.methods - MM3-2000, MMFF-94, GLYCAM, AMBER, CHARMM36, OPLS-AA, PDB.
- conformations.solvent - none, GB, STIL, TIP3P, etc.
- compounds.unit_type - chem, biol, sbiol, oligo, mono, homo, cyclo, fragment, motif.
- link_types.link_type - glycosidic, amidic, amine, diester, carbon-carbon, etc.
- disease.attr_name (attributes) - ICD code, Life stage, Sex.
- gtr.molecule role - O-antigen, CPS, EPS,core, lipid A, GPI, N-glycan, O-glycan, C-glycoside, etc.
- gtr.confirmed - *in vivo*, indirect, semi-direct, *in vitro*, *in silico*, suggested.
- publication_specific.synthesis - chemical, enzymatic, fragmentary, biosynthesis, etc.
- nmr_solvents.unit - %, vol %, M, mkM, etc.
- external_resources.resource - CA, PubChem, GlycomeDB, CCSD, US patent, GlyTouCan, etc.

The data are imported from main text dumps (see below), with a few exceptions, the detailed description of which are beyond the scope of this paper.

The conformation map subdatabase^27^ (grey headers in Fig. 2) is imported from a set of molecular dynamics files (XML, one file per molecule, described at http://csdb.glycoscience.ru/jsmol/confmap_data/processed_trajectory_format.txt) generated automatically by a dedicated postprocessor of molecular dynamics trajectories simulated by the CAT software (Conformation Analysis Tools^28^). Generation of these files is automatized and implemented at the CSDB calculation server; *ca*. 20–30 new files are completed monthly. Currently, there are 2597 data files available for download at http://csdb.glycoscience.ru/jsmol/confmap_data/minima/, including those imported from the GlycomapsDB^29^ database.

The Glycosyltransferase subdatabase (olive headers in Fig. 2) is imported from a separate set of UTF-8 text dumps that are exported from Microsoft Excel spreadsheets filled by another team of annotators. A detailed description of the glycosyltransferase dumps is beyond the scope of this paper.

Averaged chemical shifts and glycosylation effects used in the empirical NMR spectrum simulation together with a database-driven approach are stored in a set of text files, cached to memory, and used directly upon NMR simulation.

A vocabulary of supported monomeric residues, their atomic properties, stereo codes, and their records in WURCS^30^ and SMILES^31^ notations are imported from separate text files (http://csdb.glycoscience.ru/database/core/residues.txt and http://csdb.glycoscience.ru/database/core/smiles.txt).

### Annotation rules

The CSDB database is supplemented with data by means of retrospective analysis and annotation of scientific literature. The annotation procedure includes the following steps:

- ﻿Retrieval of abstracts and meta-data from the acknowledged bibliographic databases (Web of Science (Clarivate Analytics), Scopus (Elsevier), and NCBI PubMed) by using dedicated search queries (*performed by a human expert*);
- ﻿Preliminary examination of the retrieved abstracts and selection of candidate articles for annotation (*performed by a human expert*);
- ﻿Acquisition of full texts of the selected publications and secondary examination (after this stage, *ca*. 10% of the initially found papers are left for further processing) (*performed by a human expert*);
- ﻿Selection of publications containing the carbohydrate or derivative structures that match the database scope (see the criteria below) (*performed by a human expert*);
- ﻿Retrieval of the relevant information from the published data (*performed by a human annotator*);
- ﻿Encoding of the information in the strict format in a text dump file (see below) (*performed by a human annotator*);
- ﻿Various error detection routines, correction of annotation errors, and tracking of errors in publications (*performed by machine means, experts in glycobiology, and information scientists*);
- ﻿Temporary upload of the resulting dump into a service shadow of the database and subsequent checking for errors detectable in the database context only (e.g. invalid internal cross-links) (*performed by machine means and expert analysis of warnings*);
- Manual validation of the annotated data by a human curator (25–100% entries are checked);
- ﻿Approval of the dump file and its merging with the main dump, which serves as a backup of the database;
- ﻿Update of the database content from the main dump (performed annually).

To match the database scope, an article must contain at least one explicit or implicit molecular structure that meets any of the following criteria:

- ﻿The structure contains at least one carbohydrate residue (except nucleic acids studied in genomic or transcriptomic context);
- ﻿The glyco moiety of the structure is established in this or previous publications with the degree of unambiguity sufficient to derive most of its monomeric composition and at least a half of its linkages, and residue configurations;
- ﻿The structure is associated with an unambiguously specified biological source (taxon), and this taxon belongs to prokaryotes, plants, fungi or protista.

The carbohydrate structure can be published explicitly (as a figure, scheme, IUPAC name, etc.) or implicitly (as a trivial name or even a free-text description by the authors). The structure is considered present in a publication if any of the following conditions is met:

- The primary structure or its conformation is elucidated;
- A motif of the structure is suggested;
- Various properties of the structure, including its biological activity, are studied;
- Synthesis or modeling of the structure is described;
- The structure is reassigned to another taxon;
- The biological role or other properties of the structure are referenced or reviewed.

The association of a given structure with a biological source (taxon) implies any of the following:

- The structure was extracted from a biological source (i.e. the structure is natural);
- The structure is a part of a larger natural molecule, and this part is discussed separately (e.g. O-glycan moiety of a glycoprotein);
- The structure is synthetic and is identical to a natural structure (or differs from it only by an aglycon);
- The structure was obtained as a sample by modification or degradation of a natural structure, e.g. as a result of the analytical procedures;
- The structure was produced outside the organism by an enzyme from this organism, and: (i) was reported elsewhere to be present in this taxon; or (ii) its precursor was reported elsewhere to be present in this taxon or to be consumed by this taxon; or (iii) its precursor was reported elsewhere to be present in the host organism infected by this taxon.

### Text dump format

Except for derived content, such as oligosaccharide conformation maps, the CSDB is imported from human-readable text files called “dump files”. The main CSDB dump file is manually filled and appended by a team of annotators, who perform the search for matching scientific literature and its analysis. Before import, the dumps undergo automated syntactic validation and manual data quality control by another team of curators. Data correction and content updates are performed on the main dump file.

The main CSDB dump file is a UTF-8 text backup of the database and a reference file for all the database content. The dump file contains records separated by two blank lines; the main dump contains all the CSDB records. Lines starting with the symbol # are comments for annotators and are not processed. Every record is a unique combination of a molecular structure and a paper, in which this structure is discussed. These data are appended by other annotations, such as biological context, etc. The record consists of 44–47 lines, one line per field. The line starts with the field name followed by colon (**:**), after which the field content is provided. Line breaks inside the field are not allowed. The detailed explanation of the fields is given in Online-only Table 1.

As an example, we provide a step-by-step description of the annotation procedure for one of the CSDB records (ID 4676; http://csdb.glycoscience.ru/database/core/search_id.php?id_list=4676; see Online-only Table 2). This record was added to the database upon annotating the papers on the structures of carbohydrates from the bacterial genus *Proteus* that were published in the years 1996–2000. The corresponding papers were selected via the Web of Science database. The following search query can be used in the current version of the WoS (in the Advance Search mode): (TS = (carbohydrate* OR *saccharide) AND TS = (Proteus) AND PY = (1996–**2000))**. The paper itself^32^ is open-access available at the publisher web-site (https://febs.onlinelibrary.wiley.com/doi/full/10.1046/j.1432-1327.2000.01041.x).

As stated in the *Annotation rules* section, only the papers containing at least one explicit or implicit molecular structure that meets any of the above-mentioned criteria concerning the structure and its biological source are used for filling up the database. In this example, the polysaccharide structure was given in the abstract and text of the paper, and the source of this structure was unambiguously stated as *Proteus penneri* strain 63. Thus, the data from the paper were added to the CSDB database.

**Fig. 3:**
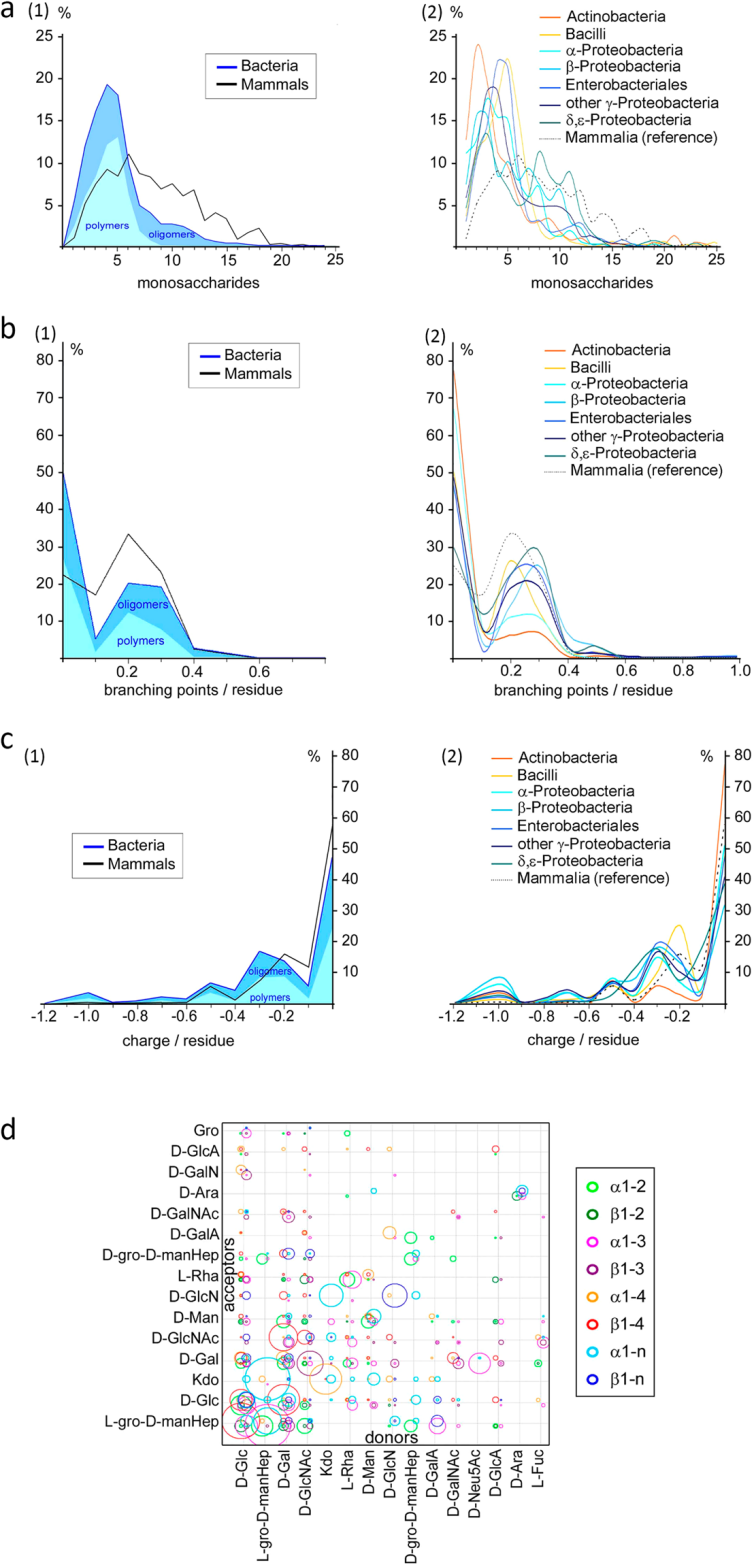
Examples of analytical studies carried out earlier directly on CSDB and Glycosciences.de content. (**a**) Size distribution of carbohydrate sequence units, (**b**) branching index distribution, and (**c**) mean charge density distribution in two taxonomic domains. (**d**) Glycosidic linkage distribution in bacterial oligomeric glycans. Reproduced with permission from^36^.

The annotation of this paper included the following steps:

1. A template for a new record was created in the text dump file. This template contained all the mandatory and optional fields (see Online-only Table 1). A CSDB ID was assigned to the record in accordance with the previously assigned IDs.
2. The bibliographical fields of the annotation form were completed: AU (authors), TI (title), JN (journal), PY (publication year), VL (volume and issue), PG (pages), and RL (bibliographical identifiers). The additional fields EA (corresponding author e-mail), AD (author affiliations), AB (abstract) and KW (keywords) were also completed. (See the corresponding fields in Online-only Table 2.) The data added to the text dump file were retrieved from page 601 of the paper.
3. The structure-related fields ST1 (the carbohydrate structure in the CSDB Linear encoding, according to the paper; the rules of the CSDB Linear encoding are beyond the scope of this paper paper and were published elsewhere^33^), ST2 (type of the structure; in this case – CHEM, chemical repeating unit of a polymer, because the exact polymerization frame, i.e. a biological repeating unit, was not reported in the paper), SL (structure location in the paper, in this case – abstract or Fig. 3 in the paper) and CC (compound classes/roles) were completed. The fields ST3 (polymerization degree), AG (aglycon information), MF (molecular formula), and 3D (3D structure and conformational data) were irrelevant or unknown and were therefore left empty. (See the corresponding fields in Online-only Table 2.)
4. The fields related to the biological source of the structure were completed: SO (the organism, from which the structure was extracted; in this case - *Proteus penneri* 63), KD (taxonomic domain/taxonomic phylum; in this case - bacteria/Proteobacteria), and TAX (identifier from the NCBI Taxonomy database; in this case - (102862), which refers to the Proteus penneri species; it is given in parentheses, because there is no separate record for strain 63 in the NCBI Taxonomy, but a TaxID exists for a higher rank on the tree of life, namely species). The additional field DSS (disease of the host organism associated with the structure or its biological source) was also completed in accordance with the International Classification of Diseases, version 11.
5. The fields related to the elucidation of the structure were completed: MT (methods, in accordance with the Materials and Methods section of the paper), NMRH (^1^H NMR assignment), NMRC (^13^C NMR assignment), NMRS (solvent, in which NMR spectra were recorded, as stated the Materials and Methods section), and NMRT (temperature, at which NMR spectra were recorded, in Kelvins, as stated in the Materials and Methods section). (See the corresponding fields in Online-only Table 2.) Templates for the fields NMRH and NMRC can be generated by using the “generate NMR template” link at the “Submit record” page in the “Extras” section at the CSDB web-site. Note that to generate an NMR template, the ST1 field must be completed.
6. In accordance with the methods given in the previous step, the TH field was completed. In this case, it contains “1”, because the paper describes the elucidation of the structure being annotated.
7. Finally, the annotator and service fields were completed: U1 (annotator’s last name), U2 (record submission date), U3 (ID of the paper in a local database of the Carbohydrate Chemistry Lab, N.D. Zelinsky Institute of Organic Chemistry, RAS), RR (IDs of related CSDB records), and DB (references to other structure databases; in this case – ID of the structure in the GlyTouCan database). (See the corresponding fields in Online-only Table 2.)

Note that in this record, several fields remain empty. Thus, the paper provided no information on the host organism and organ/tissue, from which the structure was extracted (fields HO and OTI), on the enzymes processing the structure (EI), on its biological activity (BA), biosynthesis (BG), and chemical synthesis (SY). There is also no trivial name of the compound (NC). Online-only Table 2 shows the final CSDB record 4676, as present in the CSDB text dump file.

For annotation, we use full texts of articles from publisher web sites (open-access papers), the Zelinsky Institute subscriptions and library, and requests to the authors if allowed by a publisher license. We do not provide the access to full texts themselves.

## Data Records

**Fig. 4:**
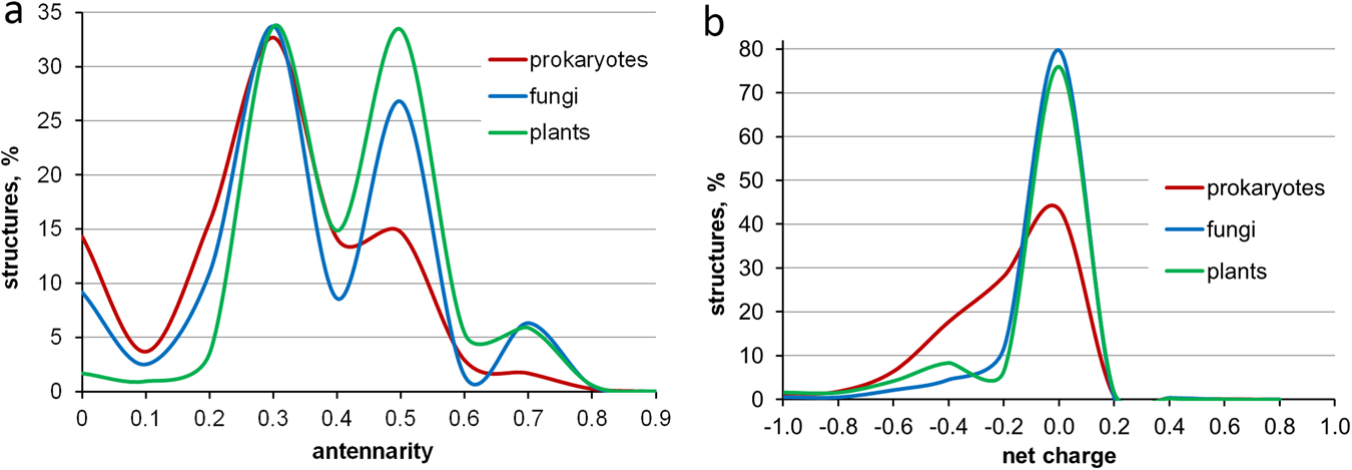
Distribution of structures in CSDB according to their antennarity (**a**) and net charge (**b**) in the organisms from the three kingdoms represented in CSDB. Prokaryotes and fungi have a complete coverage on published carbohydrate structures (up to the year 2020), while plants are covered up to 1997 only.

The following data described in this paper are publicly available^34^:

Text dump for prokaryotic carbohydrates: bcsdb_2021dec06.txt;

Text dump for fungal carbohydrates: fcsdb_2021dec06.txt;

Text dump for plant carbohydrates: pcsdb_2021dec06.txt;

Self-contained CSDB backup (for import using MySQL): CSDB_2021dec23_full.sql;

Supplementary data (Tables S1 and S2) used to prepare plots in Fig. 4;

Supplementary code used for generation of data in Tables S1 and S2 (needs a running instance of the CSDB database).

The text dump files are subject to update biennially.

## Technical Validation

The dump files are subject to machine error checking upon import. The quality of the CSDB data is maintained by automatic detection of *ca*. 100 types of data errors and suspicious data combinations. Manual verification of the data is performed by human experts; it allows revealing logical and factual errors that cannot be detected automatically^33^. The most widespread type of errors found in CSDB are those imported from other databases, e.g. CCSD (which, according to a retrospective analysis, contains errors in *ca*. 35% entries^35^).

According to manual error checking of the CSDB dumps, *ca*. 2000 errors imported from other databases (primarily CCSD) and *ca*. 350 errors in structures and NMR spectrum assignment in original publications were found. In the latter case, when the errors could be corrected without additional experimental studies, corrections were suggested. At that, the original erroneous structure was stored in the ST1ORIG field. This field is also provided when a particular structure is revised in later publications. Users can send an error correction request via a dedicated from (a link to this form is available for each database entry at the CSDB web site).

## Usage Notes

The CSDB content has been directly used for analyzing distributions of carbohydrate structures from bacteria, protista, archaea, fungi, and plants according to various criteria. Such analysis cannot be performed by means of the CSDB web interface. For example, a comparison of bacterial and mammalian carbohydrates from the viewpoint of their characteristics and diversity, in particular, structure size, branching index and mean charge density distributions (Fig. 3a–c), formalized the differences in basic features of the carbohydrate architecture between bacteria and mammals^36^. A distribution of glycosidic linkages in oligomeric (Fig. 3d) and polymeric prokariotic glycans was also analyzed. These data, in turn, were purposed for further revealing of the immunogenic patterns of pathogenic bacteria.

For illustrative purposes, we carried out an analysis of the current distribution of carbohydrate structures in CSDB in accordance with their antennarity (Fig. 4a) and net charge (Fig. 4b). In total, *ca*. 25400 structures were considered. In this work, antennarity is the ratio of the number of non-reducing termini to the number of residues in a given structure. The net charge of a molecule is a ratio of the formal integer charge of a structural unit (an oligoglycan or a repeating unit of a polymer) to the size of the structural unit. It allows estimation of the density of charged groups (such as -NH_3_^+^, -COO^−^, -PO_4_^3−^, and -SO_4_^2−^) in a glycan. A detailed description of the sampling and deriving of the statistic data is provided in Tables S1 and S2 (see the file *analysis_on_raw_DB.xls* in a dataset^34^). These two examples visually demonstrate differences and similarities between prokaryotic and eukaryotic organisms in terms of their carbohydrate architecture. Due to a close-to-complete coverage on published carbohydrate structures from bacteria and fungi, the presented distributions provide valid scientific information on the studied glycans from these organisms.

Of note, the Data Records include a self-contained CSDB image; however, we would like to note that the usage of this image is less flexible for utilizing the data being reported since it implies the same database format as the one already implemented in CSDB.

## Data Availability

The SQL and PHP code used for producing the exemplary distributions shown in Fig. 4 is provided in the file *supplementary_code_for_article.zip* and is publicly available^34^.

## Online-only Tables

**Online-only Table 1:** CSDB text dump file format.

Field^a^	Explanation
ID:	Unique permanent CSDB record ID. Records that have some unresolved problems are marked by star (*****); they are not processed until corrected. Records associated with publications that contain no information on a natural carbohydrate structure are marked with two stars (**) and are supplied with an explanation why this record is excluded from the database (e.g. **ID: 100 ** structure not found**; such records are not processed at all).
TH:	**1** if the structure is elucidated or revised in the publication (even by inaccurate methods);
	**0** if the structure discussed in the publication is elucidated elsewhere (e.g. this publication is a review or biological study of a known structure).
AU:	Comma-separated list of authors. National characters are supported (e.g. **Müller AB**).
TI:	Title of publication (article, chapter, book, symposium thesis).
JN:	Journal name without abbreviations, following the NLM standard^38^. For book publications, the following format is used: **JN: BOOK:** book name **(series:** series name, if known; otherwise, these parentheses are omitted**); Eds.:** comma-separated editors**;** Publisher. For symposium theses, the following format is used: **JN: SYMP:** symposium name **(**symposium number: year: place**); Eds.:** comma-separated editors**;** Publisher. If editor/publisher is unknown, only semicolons are typed, e.g. **SYMP: Eurocarb (17th: 2013: Tel-Aviv);;**
PY:	Publication year.
VL:	Volume number. If the volume imprint contains a subvolume/issue, e.g. **36(5)**, the subvolume/issue is given in parentheses. For books, the volume number is given outside the parentheses and the chapter number is inside the parentheses. The book series number should be included in the book title (JN).
PG:	Hyphen-separated start and end page numbers. If the end page is unknown, only the start page is provided. For imprints with an article number instead of page numbers, the ID keyword is used, e.g. **ID 35**.
RL:	References to bibliography: comma-separated list of resource-identifier pairs (resource identifier is given before the colon, reference is after the colon, e.g. **PMID:123456789**). Allowed resources are: **PMID** (NCBI PubMed ID), **DOI** (DOI code), **URL** (www address), **NLMID** (NCBI NLM ID of a book or a chapter).
EA:	E-mail of the corresponding author, e.g. **address@gmail.com; My Name <my_email@my_server.ru>**
AD:	Semicolon-separated list of author affiliations (institution, city, country). Each affiliation is listed only once; the order is arbitrary.
AB:	Publication abstract. National characters are supported. All chemical structures are either specified in a linear form or replaced with the word **/structure/**.
ST1:	Chemical structure in CSDB Linear encoding^33^. If **Subst** is used for a fully defined substructure, the corresponding SMILES code is supplied after the second equal mark, e.g. aDRibf(1–3)Subst // Subst = questin = SMILES CC1=CC(=C2C(=C1)C(=O)C3=C{3}C(=CC(=C3C2=O)OC)O)O. Please note that SMILES describes a complete residue, including hydroxy or another group that is removed upon formation of the bond to the carbohydrate moiety. The atoms that form bonds with other residues are indicated by figure brackets and the atom number (**{3}** in this example) before the corresponding carbons.
ST1ORIG^b^:	Originally published erroneous chemical structure in CSDB Linear encoding (the field is present only if ST1 contains the revised structure).
ST2:	Structure type: **OLIGO** (oligomeric structure), **CHEM** (chemical repeating unit of a polymer), **BIOL** (proven biological repeating unit), **SBIOL** (suggested biological repeating unit), **MONO** (oligomeric structure with a single carbohydrate entity), **HOMO** (repeating unit of a homopolymer), **CYCLO** (repeating unit of a cyclic polymer), **FRAGMENT** (poly- or oligomeric fragment of a larger structure), **MOTIF** (supposed, idealized or arbitrary structure; exemplary structure with certain pattern of side chains, where multiple interpretations are possible; exemplary structure with explicit values of n/m/k/etc. indicating the polymericity of subfragments (in this case, the corresponding comment is added, e.g. **NT: motif of polymeric structure 29 when n = m = 1, see ID #####**)).
ST3:	For polymers: polymerization degree preceded by **n = **, e.g. **n = 12**–**15**, or molecular mass in daltons. Ranges and relations are supported, e.g. **10000**–**30000** or >**30000**.
	For oligomers: molecular mass with or without ion type in square brackets, e.g. **9813 [M** + **H]+**.
	Multiple values are separated by commas.
SL:	Structure location in the publication, e.g. structure 1, HPLC fraction 2, compound 7a, Fig. 10, p. 456. In addition to the structure itself, tables with the NMR assignment are specified.
AG:	Aglycon information (what is attached to the reducing end of the carbohydrate structure and by which position, if known), e.g. **(−>6′) lipid A** or **inner core, bDGalpN C6**. If the aglycon is a single residue present in the CSDB monomeric space (e.g. **Allyl**) or more than one residue is attached to the aglycon, it is encoded in the structure (ST1). The AG field is used for describing aglycons if the aglycon structure could not be fully determined or when the aglycon caps the reducing end of a polymeric glycan. In all the other cases, the aglycon is encoded as a Subst alias, and its SMILES code is provided in the explanation (see the example above in ST1). The leading parentheses indicate the attachment site in the aglycon (e.g. **(−>3) sapogenin F1**). Greek letters and single and double quotes are allowed.
MF:	Molecular formula (for mono- and oligosaccharides only). First carbons, then hydrogens, then alphabetically.
NMRH:	^1^H NMR assignment data. If the spectrum is published but chemical shifts are not picked, the field is: **present in publication**.
	The proton enumeration within the residues follows the carbon enumeration. If a certain position have no protons, a hyphen (**−**) is used at the corresponding position in the proton spectrum. If a carbon has non-exchangeable protons but the proton chemical shift is not observed, a question mark (**?**) is used. Chemical shifts of two protons attached to the same carbon are separated by hyphen in the ascending order. Protons attached to a heteroatom are provided only if this heteroatom has a number in a carbon sequence, e.g. two parts of a carbon skeleton are connected via an -NH- group. Exchangeable protons (-OH, -COOH, -NH_2_, etc.) are always omitted.
	The spectrum corresponds to the exact structure specified in ST1. If the structure contains non-stoichiometric moieties (residues preceeded by **%**), spectra for the structure variant with these moieties attached are provided where possible.
NMRC:	^13^C NMR assignment data. If the spectrum is published but chemical shifts are not picked, the field is: **present in publication**. ﻿ The carbon enumeration rules are provided at the CSDB Monomer namespace web page (http://csdb.glycoscience.ru/database/core/residues.php). For residue aliases (**Subst**, etc.), the atom enumeration is explained in NT (according to IUPAC or according to the structure presentation in the paper, etc.) and follows the aglycon subdatabase (http://csdb.glycoscience.ru/database/core/aglycons.php). For unresolved chemical shifts, a question mark (**?**) is given at the corresponding positions.
	The spectrum corresponds to the exact structure specified in ST1. If the structure contains non-stoichiometric moieties (residues preceeded by **%**), spectra for the structure variant with these moieties attached are provided where possible.
NMRS:	Solvent, in which NMR experiments were carried out (chemical formula or abbreviation). Mixture components are separated with slash (e.g. **CD3OD / D2O**), starting from the solvent with the greater part. If the ratios are known, they are provided before the solvents: **90%D2O / 10%H2O / 25 mM NaCl** or **vol 67%CDCl3 / vol 33%DMSO-d6**, etc. If a reference standard is not TMS, it is specified as an additional solvent: **D2O / DSS**. pH value can be provided after semicolon: **D2O; pD 7.5**.
NMRT:	Temperature, at which NMR experiments were carried out, in Kelvins. If carbon and proton spectra were recorded at different temperatures, the values are separated with comma and the nuclei are specified in parentheses, e.g. **313(H), 298(C)**.
SO:	Semicolon-separated list of biological sources of the structure, without abbreviations. The first word in every source is interpreted as a genus, the second is for species, and all the other words (third and subsequent ones) are combined subspecies, serogroup, and/or strain.
	If a taxon was renamed or an organism was reclassified after the data had been published, the newer name is given in curly brackets after the organism, e.g. **Enterobacter sakazakii O6 {NEW: Cronobacter sakazakii O6}**. Older synonyms are specified as **{OLD:….}**. The published taxon name is always outside the curly brackets; any number of OLD and/or NEW terms can be combined inside the curly brackets.
	If the species name is unknown, while the strain is known, the species name is specified as **sp**. If only genus or genus/species information is available, incomplete taxonomic annotations are allowed. If only a rank higher than genus is known (e.g. family), is it given in parentheses. For hybrid organisms, two taxa are combined with a star (e.g. **A * B** or **A-new * B {OLD: A-old * B}**).
	In the third part of the taxon name, the following order and abbreviations for space-separated subdivisions are used: subspecies (**ssp**.), serovar (**sv**.), pathovar (**pv**.), biovar (**bv**.), serogroup (value without prefix, e.g. **O1**), strain (value or space separated values without prefixes), mutant (in parentheses, e.g. **(ΔgalE mutant)**). The strain can be a collection identifier (collection name and strain ID, e.g. **ATCC 123**), as well as a recognized or authors’ designation (e.g. **ABC123** or **Nagasaki**).
	The ranks higher than subspecies must comply with the NCBI Taxonomy^39^ naming.
	*Single taxon examples:*
	**(bacteria)** - only kingdom is known;
	**Proteus** - only genus is known;
	**Proteus penneri** - only genus and species are known;
	**Proteus penneri O22** - genus, species, serogroup;
	**Acinetobacter haemolyticus ATCC 19606** - genus, species, strain from the ATCC collection;
	**Escherichia coli O86:B7 ATCC 12701** - genus, species, serogroup, strain;
	**Citrobacter frendii PCM1555 (ΔgalE mutant)** - genus, species, mutant strain;
	**Salmonella enterica ssp. enterica sv. Typhimurium TV119** - genus, species, subspecies, serovar, strain;
	**Haemophilus parasuis sv. 5 Nagasaki** - genus, species, serovar, named strain or other subdivision;
	**Pseudomonas sp. WAK-1** - species is uknown, genus and strain are known.
KD:	Before slash (**/**): taxonomic domain (**bacteria, protista, archaea, fungi, algae, plant, animal, mammal, human**). After slash (**/**): taxonomic phylum. If there are multiple organisms of different domains or phyla, the values are separated with comma (in this case, the number of values corresponds to the number of organisms, including redesignations in curly brackets).
HO:	Systematic name (genus and species) of the host organism, in which the microorganism (specified in SO) was found, according to NCBI Taxonomy^39^ naming.
OTI:	Organ, tissue, secret, or other biomaterial, from which the structure was extracted. For microorganisms, organelle or cell part can be specified. Organs of host organisms are also allowed. If the life stage is specified (embryo, culture broth, promastigote, etc.), it is provided in this field after the **Life stage:** keyword. Only singular nouns are used; multiple entries are separated with comma; commas and parentheses within terms are not allowed.
DSS:	Disease of the host organism associated with the structure or its biological source, or disease of the patient, from which the structure was extracted. Multiple diseases are separated with semicolon; attributes are provided in square brackets when possible. A comma-separated list of attributes can include: **ICD11:** ICD-11 code, **Life stage:** life stage of the host organism, **Sex:** male or female. If no disease is known but an infectious agent has an ICD-11 code, the disease is specified as **infection due to** <taxon> **[ICD11: X**<…>**]**. Multiple diseases are not combined in one entry (like “colitis and diarrhea”). Examples: **cholera [ICD11: 1A00, ICD11: XN7N1]**; **neonatal aspergillosis [ICD11: KA63.1, ICD11: XN0WC, Life stage: neonatal]**; **infection due to Proteus penneri [ICD11: XN7PE, Sex: male]**. Only canonical disease names^40^ are used.
NC:	(Trivial) name of the compound. Greek letters, apostrophes and quotes are supported. Multiple names are separated by semicolon.
CC:	Comma-separated list of classes and roles of the compound, e.g. **O-antigen, EPS, phosphoglycolipid, GPI-anchor** etc. Not yet fully standardized vocabulary of classes is available at http://csdb.glycoscience.ru/database/core/class_method.php
MT:	Comma-separated list of methods used to elucidate or process the structure. Not yet fully standardized vocabulary of methods is available at http://csdb.glycoscience.ru/database/core/class_method.php
BA:	Biological activity of the compound (free text), including binding and serological data.
EI:	Comma-separated list of enzymes, as named by the authors, that release or process the structure, including those from other organisms, excluding the enzymes, deletions of which lead to the described (truncated) structure.
BG:	Availability of biosynthetic and genetic data in the publication (**biochemical data**, **genetic data**).
SY:	Availability of data on laboratory or industrial synthesis of the compound (**chemical**, **chemical fragmentary**, **enzymatic**, **enzymatic** ***in vivo***, **chemoenzymatic**, **chemical and enzymatic**, **synthesis** (unknown), **fragmentary** (unknown), **modeling**, **biosynthesis**).
KW:	Comma-separated list of keywords from the publication. Greek letters, apostrophes and quotes are supported.
NT:	Any comments that do not fit the other fields (e.g. errors found in the article, reference to revisions, structural elements that cannot be encoded in ST1 or AG, etc.).
3D:	Availability of 3D structure and conformation data (**conformation data**, **computer modeling**, **dynamics**, etc.).
RR:	Comma-separated list of related CSDB record IDs:
	- other structures compared with the current structure in the publication;
	- fragments of this structure;
	- similar structures with minor differences from the current structure;
	- structures of the same type from the same organism;
	- larger structures that include the current structure as their fragment;
	- other subunits of the same molecule;
	- etc.
DB:	Cross-references to other structural databases: comma-separated list of resource-identifier pairs (resource identifier is before the colon, reference is after the colon, e.g. **CA:123456789**). Widespread resource identifiers are: **CCSD** (CarbBank ID), **GlycomeDB** (GlycomeDB ID), **CA** (Chemical Abstracts access number), **CA-RN** (CA substance registry number), **US-PT** (USA patent number), **ProtDB** (Protein Databank ID), **GenDB** (GenBank ID), **GTC** (GlyTouCan ID).
TAX:	Comma-separated list of cross-references to the NCBI Taxonomy database, according to the order of organisms in the SO field. The number of values equals to the number of taxa in SO, excluding taxon redesignations in curly brackets. NCBI TaxID of the organism (strain) is provided. If no NCBI TaxID exists for the organism, NCBI TaxID of the species (or genus) is given in parentheses. If **sp**. is used for species and NCBI TaxID of the exact organism is unknown, NCBI TaxID of the genus is provided. For hybrid organisms, two TaxIDs are combined with a star (e.g. **100*101**). If a genus is missing from the NCBI Taxonomy database, TaxID of the kingdom preceded by the minus sign is provided (e.g. **−2** for bacteria). **−1** means the structure is natural but the organism is unknown.
U1:	Last name of the CSDB annotator.
U2:	Record submission date (DD.MM.YYYY).
U3:	For bacterial papers: RefManID of this paper in a local database of the Carbohydrate Chemistry Lab, Zelinsky Institute of Organic Chemistry, RAS. **PDF** is specified if a full-text PDF is present.
U4:	For bacterial papers: comma-separated list of RefManIDs of the related papers in a local database of the Carbohydrate Chemistry Lab, Zelinsky Institute of Organic Chemistry, RAS.
U5^b^:	If the record was imported from another database (e.g. CarbBank), U5 lists errors found in the original record.
U6^b^:	Filename (with extension) of the publication full text in the CSDB local cache. PMIDs (CSDB ID if no PMID is available) are used as names (**12345678.pdf** or **id_####.pdf**).

^a^Mandatory fields are shown in bold.

^b^Optional.

**Online-only Table 2:** Record ID 4676 in the text dump file format.

Field	Content
ID:	4676
TH:	1
AU:	Shashkov AS,Kondakova AN,Senchenkova SN,Zych K,Toukach FV,Knirel YA,Sidorczyk Z
TI:	Structure of a 2-aminoethyl phosphate-containing O-specific polysaccharide of Proteus penneri 63 from a new serogroup O68
JN:	European Journal of Biochemistry
PY:	2000
VL:	267(2)
PG:	601–605
RL:	PMID:10632731, DOI:10.1046/j.1432-1327.2000.01041.x
EA:	knirel@ioc.ac.ru
AD:	N.D. Zelinsky Institute of Organic Chemistry, Russian Academy Of Sciences, Moscow, Russia; Institute of Microbiology and Immunology, University of Lodz, Poland
AB:	Lipopolysaccharide of Proteus penneri strain 63 was degraded by mild acid to give a high molecular mass O-specific polysaccharide that was isolated by gel-permeation chromatography. Sugar and methylation analyses and NMR spectroscopic studies, including two-dimensional 1H, 1H COSY, TOCSY rotating-frame NOE spectroscopy, H-detected 1H,13 C and 1H,31 P heteronuclear multiple-quantum coherence (HMQC), and 1H, 13 C HMQC-TOCSY experiments, demonstrated the following structure of the polysaccharide: /structure/ where FucNAc is 2-acetamido-2,6-dideoxygalactose and PEtn is 2-aminoethyl phosphate. The polysaccharide studied shares some structural features, such as the presence of D-GlcNAc6PEtn and an α-L-FucNAc-(1→3)-D-GlcNAc disaccharide, with other Proteus O-specific polysaccharides. A marked cross-reactivity of P. penneri 63 O-antiserum with P. vulgaris O12 was observed and substantiated by a structural similarity of the O-specific polysaccharides of the two strains. In spite of this, the polysaccharide of P. penneri 63 has the unique structure among Proteus O-antigens, and therefore a new, separate serogroup, O68, is proposed for this strain.
ST1:	–6)[Ac(1–2)]aDGlcpN(1–3)[bDGlcp(1–4),Ac(1–2)]aLFucpN(1–3)[xXEtN(1-P-6),Ac(1–2)]bDGlcpN(1–2)bDGlcp(1–
ST2:	CHEM
ST3:	
SL:	Fig. 3 (upper panel)
AG:	
MF:	
NMRH:	#2,3,3,2_Ac // #2,3,3_aDGlcpN 5.05 3.98 3.73 3.72 3.95 4.06 // #2,3,4_bDGlcp 4.63 3.45 3.53 3.45 3.42 3.76–3.97 // #2,3,2_Ac // #2,3_aLFucpN 5.00 4.46 4.10 4.08 4.47 1.31 // #2,6,0_xXEtN 4.17 3.32 // #2,6_P // #2,2_Ac // #2_bDGlcpN 4.91 3.91 3.77 3.61 3.62 4.13-4.25 // #_bDGlcp 4.55 3.55 3.55 3.42 3.44 3.73-3.91 //
NMRC:	#2,3,3,2_Ac // #2,3,3_aDGlcpN 99.0 54.5 72.4 70.5 72.2 69.4 // #2,3,4_bDGlcp 104.2 75.0 77.5 70.9 76.8 61.9 // #2,3,2_Ac // #2,3_aLFucpN 98.6 50.4 71.4 79.1 68.9 16.7 // #2,6,0_xXEtN 63.2 41.3 // #2,6_P // #2,2_Ac // #2_bDGlcpN 102.2 57.0 78.9 69.5 75.9 66.0 // #_bDGlcp 103.0 81.2 77.4 70.8 76.8 61.6 //
NMRS:	D2O
NMRT:	333
SO:	Proteus penneri 63
KD:	bacteria / Proteobacteria
HO:	
OTI:	
DSS:	infection due to Proteus penneri [ICD11:XN7PE]
NC:	
CC:	O-polysaccharide
MT:	NMR-2D, GLC, GPC, methylation, GLC-MS, 1H NMR, 13 C NMR, SDS-PAGE, Western blot
BA:	
EI:	
BG:	
SY:	
KW:	2-aminoethyl phosphate,lipopolysaccharide,O-antigen,O-serogroup,Proteus penneri
NT:	
3D:	
RR:	4138
DB:	GTC:G92605ZX
TAX:	(102862)
U1:	Khalfina
U2:	01.06.2004
U3:	3884 PDF
U4:	
